# National PReCePT Programme: a before-and-after evaluation of the implementation of a national quality improvement programme to increase the uptake of magnesium sulfate in preterm deliveries

**DOI:** 10.1136/archdischild-2022-324579

**Published:** 2023-01-08

**Authors:** Hannah B Edwards, Maria Theresa Redaniel, Carlos Sillero-Rejon, Ruta Margelyte, Tim J Peters, Kate Tilling, William Hollingworth, Hugh McLeod, Pippa Craggs, Elizabeth Hill, Sabi Redwood, Jenny Donovan, Emma Treloar, Ellie Wetz, Natasha Swinscoe, Gary A Ford, John Macleod, Karen Luyt

**Affiliations:** 1 Population Health Sciences, Bristol Medical School, University of Bristol, Bristol, UK; 2 NIHR Applied Research Collaboration (ARC) West, Bristol, UK; 3 Bristol Dental School, University of Bristol, Bristol, UK; 4 Research and Innovation, University Hospitals Bristol and Weston NHS Foundation Trust, Bristol, UK; 5 Obstetrics, University Hospitals Bristol and Weston NHS Foundation Trust, Bristol, UK; 6 West of England Academic Health Science Network, Bristol, UK; 7 Medical Sciences Division, University of Oxford, Oxford, UK; 8 Translational Health Sciences, University of Bristol Medical School, Bristol, UK

**Keywords:** Neonatology, Epidemiology, Health services research

## Abstract

**Objective:**

To evaluate the effectiveness and cost-effectiveness of the National PReCePT Programme (NPP) in increasing use of magnesium sulfate (MgSO_4_) in preterm births.

**Design:**

Before-and-after study.

**Setting:**

Maternity units (N=137) within NHS England and the Academic Health Science Network (AHSN) in 2018.

**Participants:**

Babies born ≤30 weeks’ gestation admitted to neonatal units in England.

**Interventions:**

The NPP was a quality improvement (QI) intervention including the PReCePT (Preventing Cerebral Palsy in Pre Term labour) QI toolkit and materials (preterm labour proforma, staff training presentations, parent leaflet, posters for the unit and learning log), regional AHSN-level support, and up to 90 hours funded backfill for a midwife ‘champion’ to lead implementation.

**Main outcome measures:**

MgSO_4_ uptake post implementation was compared with pre-NPP implementation uptake. Implementation and lifetime costs were estimated.

**Results:**

Compared with pre-implementation estimates, the average MgSO_4_ uptake for babies born ≤30 weeks’ gestation, in 137 maternity units in England, increased by 6.3 percentage points (95% CI 2.6 to 10.0 percentage points) to 83.1% post implementation, accounting for unit size, maternal, baby and maternity unit factors, time trends, and AHSN. Further adjustment for early/late initiation of NPP activities increased the estimate to 9.5 percentage points (95% CI 4.3 to 14.7 percentage points). From a societal and lifetime perspective, the health gains and cost savings associated with the NPP effectiveness generated a net monetary benefit of £866 per preterm baby and the probability of the NPP being cost-effective was greater than 95%.

**Conclusion:**

This national QI programme was effective and cost-effective. National programmes delivered via coordinated regional clinical networks can accelerate uptake of evidence-based therapies in perinatal care.

**WHAT IS ALREADY KNOWN ON THIS TOPIC**

- Since 2015, the UK National Institute for Health and Care Excellence (NICE) has recommended administration of magnesium sulfate (MgSO_4_) for fetal neuroprotection in very preterm deliveries (24–30 weeks’ gestation).
- By 2017, only two-thirds of eligible women in England were given MgSO_4_, with wide regional variations.
- The PReCePT (Preventing Cerebral Palsy in Pre Term labour) pilot study increased uptake from 21% to 88% (2015).
- The National PReCePT Programme (NPP) aimed to increase MgSO_4_ uptake to 85% by 2020.

**WHAT THIS STUDY ADDS**

- The NPP, providing a quality improvement (QI) toolkit, regional Academic Health Science Network support and clinical backfill funding, was effective in increasing MgSO_4_ uptake in preterm deliveries.
- The NPP was highly cost-effective, generating a net monetary benefit of £866 per preterm baby and ~£3 million over the 12 months following implementation.

**HOW THIS STUDY MIGHT AFFECT RESEARCH, PRACTICE OR POLICY**

- Research evidence can take decades to become embedded in perinatal clinical practice, as was the case for antenatal steroids.
- This study shows that national, network-supported QI programmes can accelerate uptake of evidence-based therapies and promote improvements in perinatal care.
- The PReCePT model may serve as a blueprint for future interventions to improve perinatal care.

## Introduction

Since 2015, the UK National Institute for Health and Care Excellence (NICE) has recommended administration of magnesium sulfate (MgSO_4_) to women at risk of preterm birth as a core part of maternity care.^1^ MgSO_4_ is a neuroprotective treatment that reduces the risk of cerebral palsy (CP) in preterm babies,^2^ and is a highly cost-effective intervention at approximately £1 per dose and an estimated £1 million of lifetime societal savings per case of CP avoided.^3 4^ However, by 2017, only 64% of eligible women received it.^5^ High regional variations in uptake (range 49%–78%) also indicated inequalities in perinatal care.^5^

The PReCePT (Preventing Cerebral Palsy in Pre Term labour) quality improvement (QI) toolkit was developed to improve maternity staff awareness and increase the use of MgSO_4_ in mothers at risk of giving birth ≤30 weeks’ gestation. The pilot study (five maternity units) found an increase in uptake from 21% to 88% associated with the PReCePT approach.^6^ In 2018, NHS England funded the National PReCePT Programme (NPP), which scaled up this QI intervention for national roll-out. Maternity units received regional implementation support through the 15 Academic Health Science Networks (AHSNs), with the aim of increasing MgSO_4_ use to 85% by 2020. The NPP provided the PReCePT QI toolkit (preterm labour proforma, staff training presentations, parent information leaflet, posters for the unit and a learning log)^7^ to each unit (‘National PReCePT Programme Provisions’ in online supplemental file 1). Each unit had a lead ‘PReCePT champion’ midwife with 90 hours funded backfill. AHSN-level coaching and support from a regional clinical lead (obstetrician and/or neonatologist) and NPP manager were available to each unit. The NPP was launched in two tranches (May and September 2018). A nested cluster randomised trial to determine the effectiveness of standard versus enhanced support was conducted alongside the NPP.^8^

10.1136/fetalneonatal-2022-324579.supp1 Supplementary data

This study was an effectiveness and cost-effectiveness evaluation of the NPP QI intervention in increasing MgSO_4_ uptake in mothers at risk of giving birth ≤30 weeks’ gestational age. We hypothesised that it would help increase MgSO_4_ uptake beyond the expected increase due to the underlying trend rate.

## Methods

The intervention evaluated was a QI programme as described in the previous section. The method used to evaluate the intervention followed a quasi-experimental before-and-after design, comparing absolute difference in mean MgSO_4_ uptake between 12 months pre-implementation and 12 months post implementation, adjusted for possible confounders. A quasi-experimental approach was appropriate as the PReCePT intervention had been widely implemented in maternity units in England, making a standard randomised controlled trial (RCT) infeasible.^9–11^ Additionally, as there was already indication that the intervention was effective from the pilot study, sufficient clinical equipoise for an RCT with a no-intervention control group was arguably not present.

### Data

Pseudonymised patient-level data from the UK National Neonatal Research Database (NNRD) were used. This collates information from neonatal units and includes clinical data and mother and baby sociodemographic characteristics. All NNRD data undergo multiple quality assurance procedures and are considered to have high accuracy and completeness.^12 13^

Estimated NPP adoption dates for each unit, demarcating the two periods, were provided by the AHSNs. Adoption date was defined as the month when the unit had initiated an implementation plan. The total period pre-implementation and post implementation across all units covered the months between October 2017 and June 2020. The month of initiation of the NPP in the maternity unit was excluded from the analysis.

### Outcome

For consistency with nationally reported audit data, MgSO_4_ uptake was defined as the number of mothers receiving MgSO_4_ divided by the total number of eligible mothers, excluding missing values from the denominator. This was expressed as a percentage and computed per month per unit. For the cost-effectiveness analysis only, missing MgSO_4_ uptake was considered as ‘not given’ and included in the denominator.

The analysis included data on babies born ≤30 weeks’ gestational age. Singletons and one baby (the first born) from each multiple birth were included, for consistency with nationally reported figures. All multiples were included in the description of baby-level demographics. In cases where only one baby had a record for MgSO_4_, we recoded the missing MgSO_4_ status of the other multiples to match that for their twin/triplet who did have a record. For multiples with conflicting records (eg, Baby 1=given, Baby 2=not given), we recorded MgSO_4_ as given.

The secondary outcomes were trends in MgSO_4_ uptake, missing MgSO_4_ data, reasons MgSO_4_ was not given, cost of the NPP per preterm baby and the incremental net monetary benefit of the NPP per preterm baby from a lifetime societal perspective.

### Possible confounders and other model terms

Possible confounding factors adjusted for included baby birth weight (grams) adjusted for gestational age (weeks) and sex expressed as a z-score, whether the baby was part of a multiple birth, maternal age, ethnicity, level of deprivation (Index of Multiple Deprivation decile), hypertension during pregnancy, type of unit (high dependency unit or special care unit (HDU/SCU) vs neonatal intensive care unit (NICU)), time and clustering by AHSN. The cost-effectiveness analysis also adjusted for type of birth (imminent or threatened).

Mother and baby characteristics were aggregated to the maternity unit level (using non-missing information) per study month, for example, mean maternal age and proportion with pregnancy hypertension. Missing information was minimal, except for mother’s ethnicity (online supplemental table S1). The cost-effectiveness analysis used baby-level data, and missing data on possible confounders were imputed through chained equations.^14^

### Statistical analyses

#### Effectiveness analysis

To compare the difference in mean monthly MgSO_4_ uptake pre-implementation and post implementation, we conducted a multilevel mixed-effects linear regression using the maternity unit as the primary level of analysis. The model was weighted on unit size (number of eligible mothers at each unit) and adjusted for clustering by AHSN and potential confounders as listed in the previous section.

To account for early and late start of NPP activities in many units (as reported by AHSNs), we excluded records within three months either side of the NPP adoption month as a sensitivity analysis.

As additional sensitivity analyses, we evaluated the effect of (1) including the 13 maternity units receiving enhanced support in the PReCePT trial intervention arm and (2) excluding units in one AHSN that started adoption significantly later than other AHSNs.

#### Cost-effectiveness analysis

The mean implementation cost per maternity unit was estimated from data supplied by the national programme team. This included NPP management, AHSN support, and clinical backfill for midwives and clinical leads. The mean implementation cost per baby was calculated as the cost per unit divided by the total number of eligible babies per unit delivered during the follow-up period.

A decision tree analysis estimated the net monetary benefit of the NPP using a lifetime horizon and societal perspective.^15^ Model parameters were based on NNRD data for MgSO_4_ uptake, and reported estimates for lifetime gains in quality-adjusted life-years (QALYs) and societal cost savings relating to healthcare, education, housing and work productivity from preventing CP via MgSO_4_ treatment.^4^ Cost estimates were converted to pounds sterling and inflated to 2019 prices (online supplemental table S2). Babies delivered by caesarean section were defined as ‘imminent’ births (certain to occur within 24 hours) and all others as ‘threatened’. Deterministic analysis used a willingness-to-pay threshold of £20 000 per QALY gained, in line with the NICE guidance.^16^

The difference in uptake between the baseline and follow-up periods was estimated using a multilevel mixed-effects linear logistic regression at the baby level, adjusted for clustering by AHSN and unit, listed confounders, and interaction between type of birth and time period.

Probabilistic analysis to characterise parameter uncertainty and to estimate cost-effectiveness used Monte Carlo simulation with 10 000 samples drawn from the parameter distributions.^15^ For lifetime costs and health utilities estimates, we used the incremental differences. Point estimates, distribution assumptions and parameter source estimates are reported in online supplemental table S3. Incremental costs and effects were plotted on the cost-effectiveness plane and a cost-effectiveness acceptability curve plotted for willingness-to-pay thresholds from 0 to £100 000 per QALY gained. Subgroup analysis explored differences in cost-effectiveness between types of maternity unit (SCU/HDU or NICU).

## Results

Of the 155 maternity units in England, 150 participated in the NPP (the five units not participating were study pilot sites).^6^ The 13 units comprising the nested cluster RCT intervention group were also excluded, leaving 137 maternity units for evaluation.

The NPP adoption dates of the participating units ranged from October 2018 to October 2020, with almost all starting by April 2019. On average, there were 2.9 preterm births per unit per month. Maternal and baby characteristics were similar pre-implementation and post implementation (table 1). The average MgSO_4_ uptake across all units in the 12 months pre-implementation was 70.9%, increasing to 83.1% across the 12 months post implementation (table 2). The average amount of missing MgSO_4_ data reduced from 2.9% to 1.4%.

**Table 1:** Sociodemographic and clinical characteristics of mothers and babies born at ≤30 weeks’ gestation in NPP maternity units in England, October 2017–June 2020

Variable	Pre-implementation*	Post implementation*
Sociodemographic characteristics of babies†		
Babies (n)	3630	3441
Gestational age (weeks), median (IQR)	27.9 (25.9–29.0)	27.9 (26.0–29.1)
Birth weight (g), median (IQR)	982 (770–1210)	980 (769–1205)
Birth weight adjusted for gestational age (z-score), median (IQR)	0.1 (−0.6 to 0.7)	0.1 (−0.6 to 0.7)
Male sex, n (%)	1960 (54.0)	1851 (53.8)
Multiple births, n (%)	871 (24.0)	817 (23.7)
Sociodemographic and clinical characteristics of mothers		
Mothers (n)	3189	3016
Maternal age (years), mean (SD)	31 (6)	31 (6)
White ethnicity, n (%)	1711 (60.2)	1610 (61.2)
Level of deprivation (IMD quintile), n (%)		
1 (most deprived)	1108 (31.0)	1112 (32.9)
2	912 (25.6)	773 (22.9)
3	643 (18.0)	621 (18.4)
4	485 (14.0)	454 (13.4)
5 (least deprived)	422 (11.82)	418 (12.4)
Hypertension in pregnancy, n (%)	128 (3.5)	157 (4.6)
Antenatal steroids given, n (%)	3340 (92.1)	3220 (93.9)
Maternity unit characteristics		
Special care unit/high dependency unit, n (%)	1336 (36)	1226 (35.6)
Neonatal intensive care unit, n (%)	2294 (63.2)	2215 (64.4)
Average number of eligible births per hospital per month, mean (SD)	2.9 (2.1)	2.9 (2.1)

*Figures cover the 12 months prior to, and 12 months following the recorded NPP adoption date at each unit, excluding the month of adoption itself.

†All babies in the dataset including multiples

IMD, Index of Multiple Deprivation; NPP, National PReCePT Programme; PReCePT, Preventing Cerebral Palsy in Pre Term labour.

**Table 2:** MgSO_4_ uptake in babies born at ≤30 weeks’ gestation in NPP maternity units in England, October 2017–June 2020*

Variable	Pre-implementation†	Post implementation†
Total number of eligible births	3172	3014
Total number of mothers given MgSO_4_, n (%)	2279 (71.9)	2527 (83.8)
Total number of mothers not given MgSO_4_, n (%)	803 (25.3)	447 (14.8)
Total number with MgSO_4_ data missing, n (%)	90 (2.8)	40 (1.3)
Mean MgSO_4_ uptake across all units, % (SD)	70.9 (3.6)	83.1 (3.5)
Mean MgSO_4_ missing data across all units, % (SD) (% range)	2.9 (1.3) (1.1–5.6)	1.4 (1.0) (0–3.1)
Reason MgSO_4_ not given, n (%)		
Contraindicated	9 (1.2)	6 (1.3)
Declined	7 (0.7)	3 (0.7)
Delivery imminent	499 (62.1)	337 (75.4)
Not appropriate	69 (8.6)	24 (5.4)
Not offered	129 (16.1)	51 (11.4)
Data missing	90 (11.2)	26 (5.8)

*MgSO_4_ data from records on singleton births and the first born of multiples with records in the data set.

†Figures cover the 12 months prior to, and 12 months following the recorded NPP adoption date at each unit, excluding the month of adoption itself.

MgSO_4_, magnesium sulfate; NPP, National PReCePT Programme; PReCePT, Preventing Cerebral Palsy in Pre Term labour.

Imminent delivery was the most common reason why MgSO_4_ was not given. Pre-implementation, MgSO_4_ was ‘not offered’ in 16.1% of cases, and post implementation this reduced to 11.4% (table 2).

**Figure 1:**
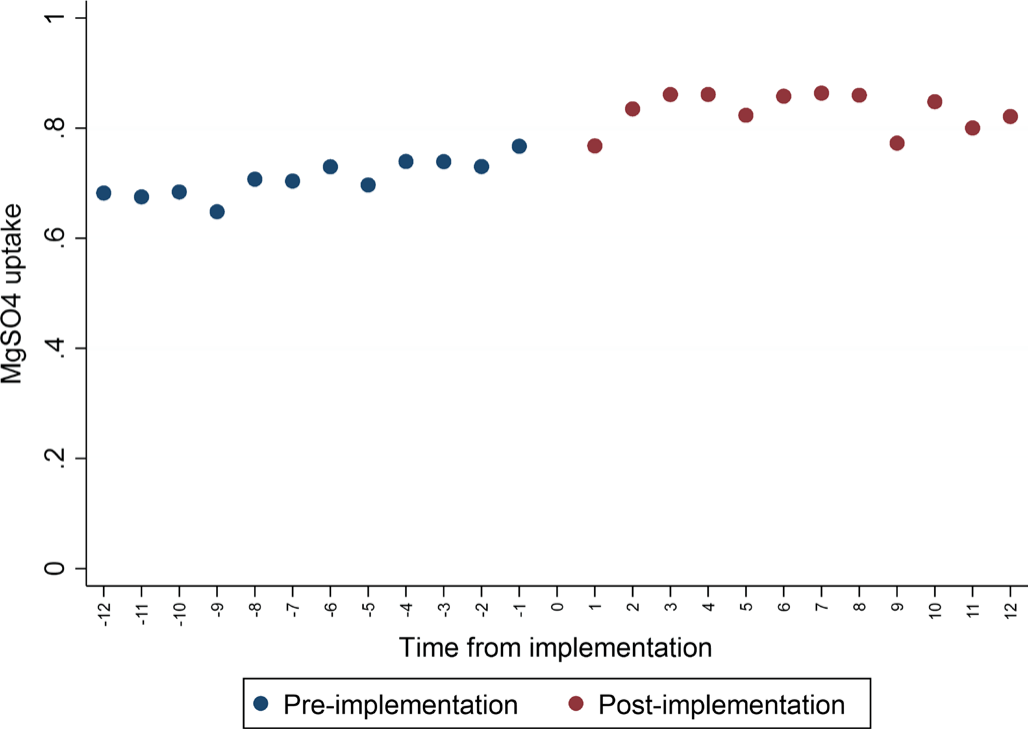
Magnesium sulfate (MgSO_4_) uptake pre-implementation and post implementation.

Overall, the trend in MgSO_4_ uptake increased steadily (figure 1, online supplemental figures S1–S3). The average uptake varied by AHSN, and within each AHSN there was high monthly variation (online supplemental figure S3). The lowest average uptake was around 65% at the end of 2017 and the highest was around 94% around May 2020.

The unadjusted average increase in uptake from pre-implementation to post implementation was 12.2 percentage points. After adjusting for confounding factors, this reduced to 6.3 percentage points (95% CI 2.6 to 10.0 percentage points, p<0.001). Sensitivity analysis excluding data three months either side of the adoption month changed the estimate to 9.5% (95% CI 4.3 to 14.7, p<0.001) (table 3). Neither including the nested RCT intervention units nor excluding units from one late-starting AHSN had any substantial effect on the estimate.

**Table 3:** Difference in MgSO_4_ uptake in babies born at ≤30 weeks’ gestation after implementation of the NPP in maternity units in England††

	Models	Difference in MgSO_4_ uptake (percentage points)*	95% CI (percentage points)	P value
1	Unadjusted†	12.2	9.5 to 15.0	<0.001
2	Adjusted for unit size‡	11.0	8.9 to 13.1	<0.001
3	Adjusted for unit size and clustering by AHSN§	11.0	8.4 to 13.5	<0.001
4	Adjusted for unit size, clustering by AHSN and NPP month¶	6.7	2.8 to 10.5	0.001
5	Fully adjusted**	6.3	2.6 to 10.0	0.001
6	Fully adjusted model** and excluding records within 3 months either side of the start month	9.5	4.3 to 14.7	<0.001
	Additional analyses			
7	Model 6 and including the 13 PReCePT trial intervention units	9.6	4.4 to 14.8	<0.001
8	Model 6 and excluding units in one AHSN where implementation was delayed	10.0	3.9 to 16.0	0.001

*Percentage point changes, post implementation minus pre-implementation.

†Crude regression of uptake post implementation compared with pre-implementation.

‡As per model 1, plus additionally weighted on the number of eligible records per unit, with robust SEs.

§As per model 2, plus additionally accounting for clustering by AHSN, with robust SEs.

¶As per model 3, plus additionally adjusted for recorded start month.

**As per model 4, plus additionally adjusted for birth weight adjusted for gestational age and sex, maternal age, IMD, ethnicity, multiple birth, maternal hypertension (all unit-level aggregates), level of unit and study month.

††MgSO_4_ data from records on singleton births and the first born of multiples with records in the data set.

AHSN, Academic Health Science Network; IMD, Index of Multiple Deprivation; MgSO_4_, magnesium sulfate; NPP, National PReCePT Programme; PReCePT, Preventing Cerebral Palsy in Pre Term labour.

The proportion of missing MgSO_4_ data fluctuated between 0% and 7%, but overall decreased over time (online supplemental figures S4 and S5). Around April 2020, the time of the first COVID-19 lockdown in England, missing MgSO_4_ data appeared to increase and uptake decrease.

### Costs and cost-effectiveness analyses

The mean implementation cost of the NPP was £6044 per unit: £738 for NPP management, £2764 for AHSN funding and £2500 for clinical backfill funding. The mean implementation cost per eligible preterm baby (≤30 weeks’ gestation) was £267.

The NPP was associated with a mean increase of 0.01 QALYs per preterm baby and £649 total incremental savings over a baby’s lifetime (table 4). This equates to a net monetary benefit of £886 per eligible preterm baby at a willingness-to-pay threshold of £20 000 per QALY gained (table 4). Applying this finding across all the preterm babies delivered during the year post implementation, the NPP was associated with savings to the society accruing over their lifetime totalling £3 million (£886 multiplied by 3441). The probability of the NPP being cost-effective was greater than 95% (table 4, online supplemental figures S6–S8).

**Table 4:** Probabilistic analysis results of the NPP cost-effectiveness

NPP	Combined			SCU/HDU			NICU		
	Point estimate	Lower 95% limit	Upper 95% limit	Point estimate	Lower 95% limit	Upper 95% limit	Point estimate	Lower 95% limit	Upper 95% limit
Incremental implementation costs, £	294	32	808	553	134	1277	98	57	149
Incremental lifetime costs, £	−943	−1486	−469	−909	−1514	−362	−867	−1443	−357
Incremental total costs, £	−649	−1284	35	−356	−1131	539	−769	−1344	258
Incremental QALYs	0.01	0.01	0.02	0.01	0.01	0.02	0.01	0.01	0.02
Net monetary benefit*, £	886	130	1621	586	−372	1475	987	355	1675

*We used a willingness-to-pay threshold of £20 000 per QALY gained.

HDU, high dependency unit; NICU, neonatal intensive care unit; NPP, National PReCePT Programme; PReCePT, Preventing Cerebral Palsy in Pre Term labour; QALYs, quality-adjusted life-years; SCU, special care unit.

Although the cost per baby was higher in smaller (SCU/HDU) units than in NICUs (table 4), the probability of cost-effectiveness in small units was still high at about 85% (online supplemental figures S6–S8).

## Discussion

This is the first evaluation of a UK universally implemented national perinatal QI programme to increase administration of an evidence-based drug. We found that the NPP increased the uptake of MgSO_4_ in babies born at ≤30 weeks’ gestation and was cost-effective. It generated an estimated net monetary benefit to the society of £3 million over the lifetime of the preterm babies delivered during the 12 months following implementation. The reduction in the amount of missing MgSO_4_ data indicates an improvement in record-keeping and is likely an indirect beneficial effect of the NPP.

MgSO_4_ uptake varies across countries, with estimates of 0%–12.3% in Europe (2011–2015)^17^ and 43.0% in Canada (2011–2015).^18^ We found one similar QI strategy (MAG-CP (MAGnesium sulphate for fetal neuroprotection to prevent Cerebral Palsy) in Canada) which included educational rounds, focus groups and surveys of barriers/facilitators, and online training in addition to national guidelines.^18^ This was associated with an absolute increase in uptake of 44.3%, from 2.0% pre-implementation (2005–2010) to 46.3% post implementation (2011–2015).^18^ For context, UK uptake was 38% in 2014.^19^

### Strengths

Routinely collected maternal, neonatal and cost data from the NNRD and the NPP provided robust, high-quality data for evaluation. Almost all maternity units in England were included, making the results generalisable. Mixed-effects models enabled the effect estimates to be adjusted for known potential confounders and clustering by AHSN. As NPP implementation was directed by AHSNs, unmeasured similarities and differences between units within AHSNs need to be taken into account as we have done here.

The National Neonatal Audit Programme report on 2020 data concurred with our conclusions.^20^ They found uptake in Scotland and Wales was comparable with the national average pre-NPP, but below afterwards. This is suggestive that English units’ exposure to the NPP was associated with their higher average uptake. Their data on improvements in other audit measures are also illuminating. Their audit shows the increase in uptake of antenatal steroids from 75.6% to 85.8% took 5–6 years, where the comparable increase in the uptake of MgSO_4_ from 72% to 85.7% took place over just 2 years. A key difference in the journey of these treatments was the dedicated national QI programme for MgSO_4_. Again this is suggestive of the NPP’s role in the relatively rapid improvement in uptake of MgSO_4_.

### Limitations

In a pragmatic before-and-after observational evaluation of this kind, it is impossible to conclusively attribute the observed increase in uptake to the NPP alone. For example, some of the observed improvements in uptake could be explained by improvements in record-keeping. However, the reduction in missing data was much smaller than the observed improvement in uptake, so it is unlikely to be a significant explanatory factor. Historically, uptake has been increasing since 2014 (online supplemental figure S2). This historical trend has been accounted for in the analysis. The estimate is the increased slope (increased improvement in uptake) over and above the pre-implementation slope. The statistical methods used minimised the impact of known biases and confounders, giving reason to believe that the NPP did have a positive impact on uptake. Analyses were limited to the available data (to June 2020), but it is expected that NPP benefits will persist. Sustainability needs to be addressed in future studies. Adoption dates used to demarcate the exposure periods were not firmly defined, and NPP activities were reported to have started before or after the stated start dates in some units, possibly diluting the observable effects of the NPP on uptake. Despite this, the various sensitivity analyses did not alter the main findings.

The adjusted effect estimate was smaller than expected from previous audit figures.^21^ This suggests that other factors (eg, organisational context^22^) could have also contributed to the observed increase in uptake. From our 4-year experience post implementation in the five pilot sites, the improved uptake is likely to be sustained, meaning that longer-term analyses may show the NPP to be even more cost-effective than estimated here as implementation costs are non-recurring.

The observed decrease in uptake and increase in missing MgSO_4_ data around April 2020 may be a random fluctuation, but is consistent with a possible impact of the first wave of COVID-19 in England. Staffing pressures of a pandemic are likely to affect the quality of care. Also, women may have presented to hospital later during this time due to caution about contact, meaning missed opportunities to give MgSO_4_ due to imminent delivery. Further analysis of future data would be valuable to identify clearer trends in uptake or missing data associated with the course of the pandemic.

### Implications for clinical practice

Uptake of new evidence or guidelines is often slow due to practical barriers, lack of knowledge, and need for behaviour change, as illustrated by the case of antenatal steroids which took decades to become embedded in routine practice. This comes at a high clinical and economic cost. The NPP demonstrates that active implementation of national initiatives using QI toolkits, clinical leadership and regional QI support can have a substantial effect on accelerating uptake of evidence-based therapies.

## Data Availability

Anonymised individual-level data for this study are from the NNRD. Our data sharing agreement with the NNRD prohibits sharing data extracts outside of the University of Bristol research team. The NNRD data dictionary is available online and copies of the Statistical analysis plan are available at the University of Bristol’s institutional repository ( https://research-information.bris.ac.uk/en/projects/national-precept-prevention-of-cerebral-palsy-in-pre-term-labour- ).

## References

[1] National Institute for Health and Care Excellence (NICE) guideline NG25 . Preterm labour and birth NG25. UK NICE; 2015. 37184164

[2] Doyle LW , Crowther CA , Middleton P , et al . Magnesium sulphate for women at risk of preterm birth for neuroprotection of the fetus. Cochrane Database Syst Rev 2009;1:CD004661. 10.1002/14651858.CD004661.pub3 17636771

[3] Shih STF , Tonmukayakul U , Imms C , et al . Economic evaluation and cost of interventions for cerebral palsy: a systematic review. Dev Med Child Neurol 2018;60:543–58. 10.1111/dmcn.13653 29319155

[4] Bickford CD , Magee LA , Mitton C , et al . Magnesium sulphate for fetal neuroprotection: a cost-effectiveness analysis. BMC Health Serv Res 2013;13:527. 10.1186/1472-6963-13-527 24350635 PMC3878233

[5] NNAP . National neonatal audit programme (NNAP) annual report 2017. UK RCPCH; 2018.

[6] Burhouse A , Lea C , Ray S , et al . Preventing cerebral palsy in preterm labour: a multiorganisational quality improvement approach to the adoption and spread of magnesium sulphate for neuroprotection. BMJ Open Qual 2017;6:e000189. 10.1136/bmjoq-2017-000189 PMC5699159 29450301

[7] AHSN . Precept national programme resources. Available: http://www.ahsnnetwork.com/about-academic-health-science-networks/national-programmes-priorities/precept/precept-resources

[8] Edwards HB , Redaniel MT , Sillero-Rejon C , et al . Evaluation of standard and enhanced quality improvement methods to increase the uptake of magnesium sulphate in pre-term deliveries for the prevention of Neurodisability (PRECEPT study): a cluster randomized controlled trial. medRxiv 2022. 10.2139/ssrn.4071359 37691262

[9] Bernal JL , Cummins S , Gasparrini A . Interrupted time series regression for the evaluation of public health interventions: a tutorial. International Journal of Epidemiology 2016;46:348–55. 10.1093/ije/dyw098 PMC5407170 27283160

[10] Craig P , Katikireddi SV , Leyland A , et al . Natural experiments: an overview of methods, approaches, and contributions to public health intervention research. Annu Rev Public Health 2017;38:39–56. 10.1146/annurev-publhealth-031816-044327 28125392 PMC6485604

[11] de Vocht F , Katikireddi SV , McQuire C , et al . Conceptualising natural and quasi experiments in public health. BMC Med Res Methodol 2021;21:32. 10.1186/s12874-021-01224-x 33573595 PMC7879679

[12] Battersby C , Statnikov Y , Santhakumaran S , et al . The United Kingdom national neonatal research database: a validation study. PLoS One 2018;13:e0201815. 10.1371/journal.pone.0201815 30114277 PMC6095506

[13] Gale C , Morris I . The UK national neonatal research database: using neonatal data for research, quality improvement and more. Archives of disease in childhood - Education &amp; practice edition 2016;101:216–8. 26968617 10.1136/archdischild-2015-309928 PMC4975807

[14] Lee KJ , Tilling KM , Cornish RP , et al . Framework for the treatment and reporting of missing data in observational studies: the treatment and reporting of missing data in observational studies framework. J Clin Epidemiol 2021;134:79–88. 10.1016/j.jclinepi.2021.01.008 33539930 PMC8168830

[15] Drummond MSM , Claxton K , Stoddart G . Methods for the economic evaluation of health care programmes. 4th ed. Oxford: Oxford University Press, 2015.

[16] NICE . Developing NICE guidelines: the manual. London: NICE, 2014.

[17] Wolf HT , Huusom L , Weber T , et al . Use of magnesium sulfate before 32 weeks of gestation: a European population-based cohort study. BMJ Open 2017;7:e013952. 10.1136/bmjopen-2016-013952 PMC5278293 28132012

[18] De Silva DA , Synnes AR , von Dadelszen P , et al . MAGnesium sulphate for fetal neuroprotection to prevent Cerebral Palsy (MAG-CP)-implementation of a national guideline in Canada. Implement Sci 2018;13:8. 10.1186/s13012-017-0702-9 29325592 PMC5765609

[19] Lea CL , Smith-Collins A , Luyt K . Protecting the premature brain: current evidence-based strategies for minimising perinatal brain injury in preterm infants. Arch Dis Child Fetal Neonatal Ed 2017;102:F176–82. 10.1136/archdischild-2016-311949 28011793

[20] NNAP . National neonatal audit programme (NNAP) annual report 2020. UK RCPCH; 2021.

[21] NNAP . National neonatal audit programme (NNAP) annual report 2018. UK RCPCH; 2019.

[22] Bain E , Bubner T , Ashwood P , et al . Barriers and enablers to implementing antenatal magnesium sulphate for fetal neuroprotection guidelines: a study using the theoretical domains framework. BMC Pregnancy Childbirth 2015;15:176. 10.1186/s12884-015-0618-9 26283623 PMC4539663

