# Supplementary material for: National PReCePT Programme: a before-and-after evaluation of the implementation of a national quality improvement programme to increase the uptake of magnesium sulfate in preterm deliveries
Source: Arch Dis Child Fetal Neonatal Ed. 2023 Jan 8;108(4):342–7. doi: 10.1136/archdischild-2022-324579 (PMC10314002; doi:10.1136/archdischild-2022-324579)
Supplement: Supplementary data [file fetalneonatal-2022-324579supp001.pdf]

Supplementary Material

Contents

National PReCePT Programme provisions 2
Table S1. Missing data on possible confounders, aggregated at maternity unit level 3
$MgSO_4$ uptake through time in maternity units in England 4
Figure S1. $MgSO_4$ uptake through time in maternity units in England, October 2017 – June 2020 4
Figure S2. MgSO4 uptake through time in maternity units in England, January 2014 – June 2020 5
Figure S3. MgSO4 uptake by AHSN*, October 2017 – June 2020 6
Trend in missing $MgSO_4$ data over time in maternity units in England 9
Figure S4. Trend in missing $MgSO_4$ data over time, October 2017 – June 2020 9
Figure S5. Trend in missing MgSO4 data over time, January 2014 – June 2020 10
Economic evaluation 11
Table S2. Estimated lifetime costs and QALYs per patient associated with MgSO4 treatment† 11
Table S3. Point estimates, probability distributions, and source of parameter estimates used in the probabilistic analysis 12
Table S4. Mean lifetime QALYs and costs per baby by type of birth and trial arm 13
Table S4. Deterministic Analysis Results of the NPP cost-effectiveness 14
Figure S6. Cost-effectiveness plane of National PreCePT Programme 15
Figure S7. Cost-effectiveness plane of National PreCePT Programme for SCU/HDU units 16
Figure S8. Cost-effectiveness plane of National PreCePT Programme for NICU units 17
Figure S9. Cost-effectiveness acceptability curve of National PreCePT Programme 18
Figure S10. Cost-effectiveness acceptability curve of National PreCePT Programme for SCU/HDU units 19
Figure S11. Cost-effectiveness acceptability curve of National PreCePT Programme for NICU units 20

Edwards HB, *et al. Arch Dis Child Fetal Neonatal Ed* 2023; 108:342–347. doi: 10.1136/archdischild-2022-324579

National PReCePT Programme provisions

Item	Definition
PReCePT QI toolkit	Clinical guidance;<br>Pre-term labour proforma template;<br>Staff training presentations;<br>Parent leaflet;<br>Posters for display on the unit to raise staff awareness;<br>A QI Learning Log;<br>Project Dashboard;<br>Pens, magnets, lanyards and other aide-mémoires to promote $MgSO_4$ to unit staff (if purchased)
QI training	Local level (AHSN or unit level) QI training and guidance to adapt materials for local use, cascaded from AHSN
Regional support	Support from a AHSN level clinical lead (obstetrician and neonatologist) and AHSN lead
Local clinical champion	Local obstetrician and neonatologist identified by unit to guide and oversee local implementation
Funded time for local midwife champion	Funded time of up to 90 hours per unit (on average 2 hours per week)
National support	AHSN Network steering group;<br>National lead and manager;<br>Shared learning events between AHSN managers leading the NPP in their area

Table S1. Missing data on possible confounders, aggregated at maternity unit level

Variable	Number missing	Proportion missing[0]
Birthweight	2	0.03%
Multiple birth	1	0.01%
Maternal age	37	0.52%
Mother's ethnicity	1559	22.61%
Level of deprivation (IMD decile)	123	1.74%

[0]out of 7071 aggregated maternity unit level data points

$MgSO_4$ uptake through time in maternity units in England

Figure S1. $MgSO_4$ uptake through time in maternity units in England, October 2017 – June 2020

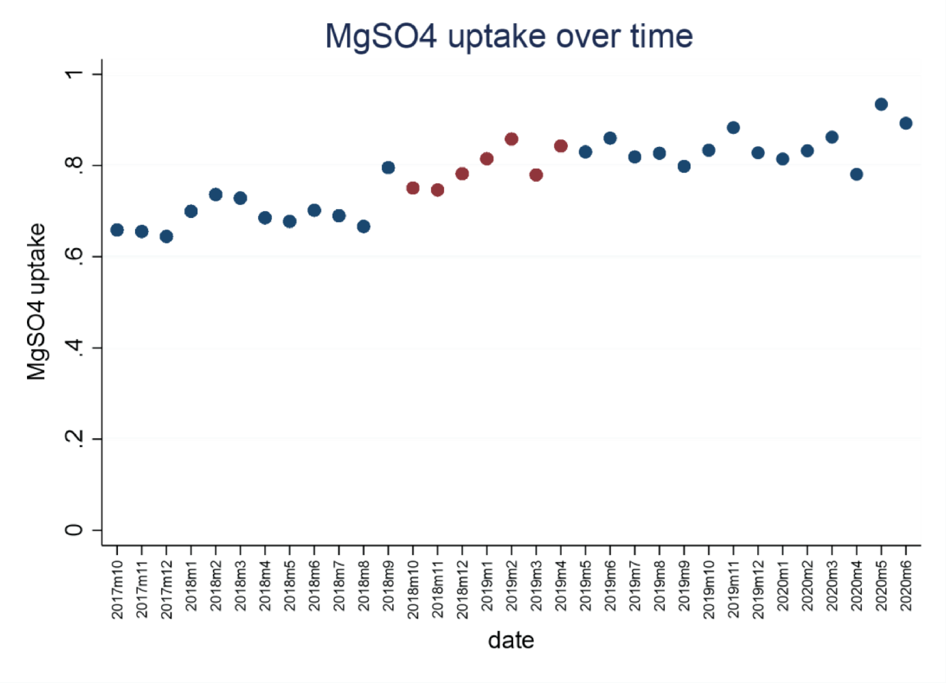

● Pre- or post-implementation periods ● NPP implementation start dates

Figure S2. MgSO4 uptake through time in maternity units in England, January 2014 – June 2020

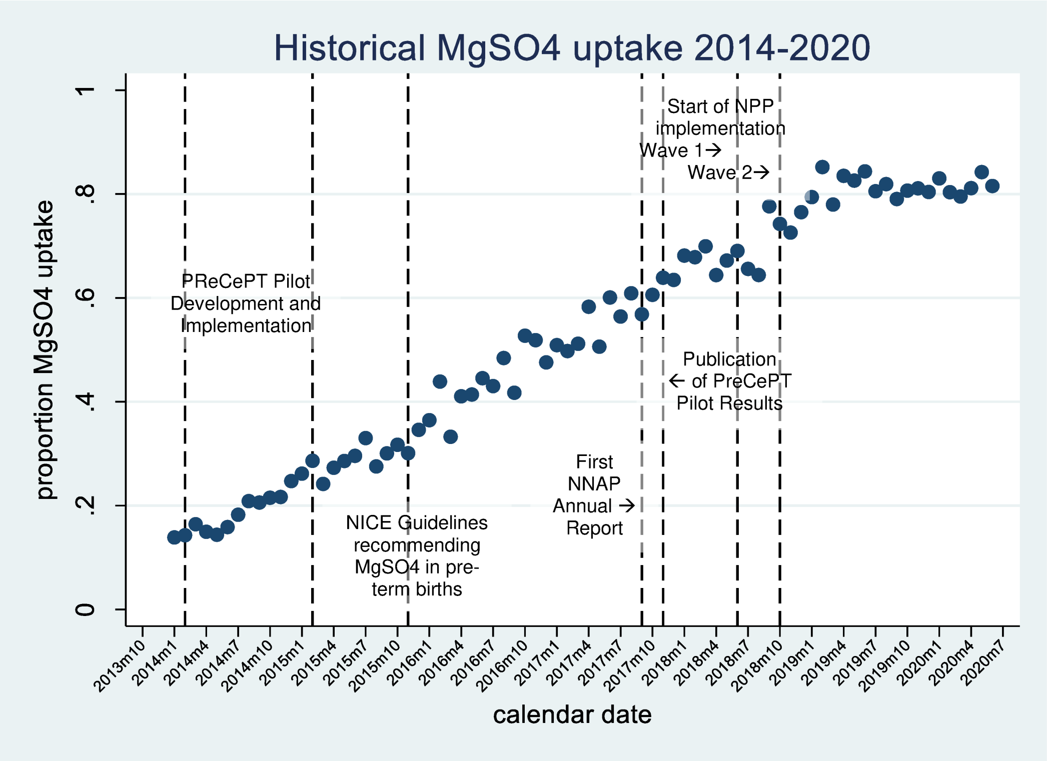

Figure S3. MgSO4 uptake by AHSN*, October 2017 – June 2020

● Pre-implementation periods ● Post-implementation period (including NPP start date)

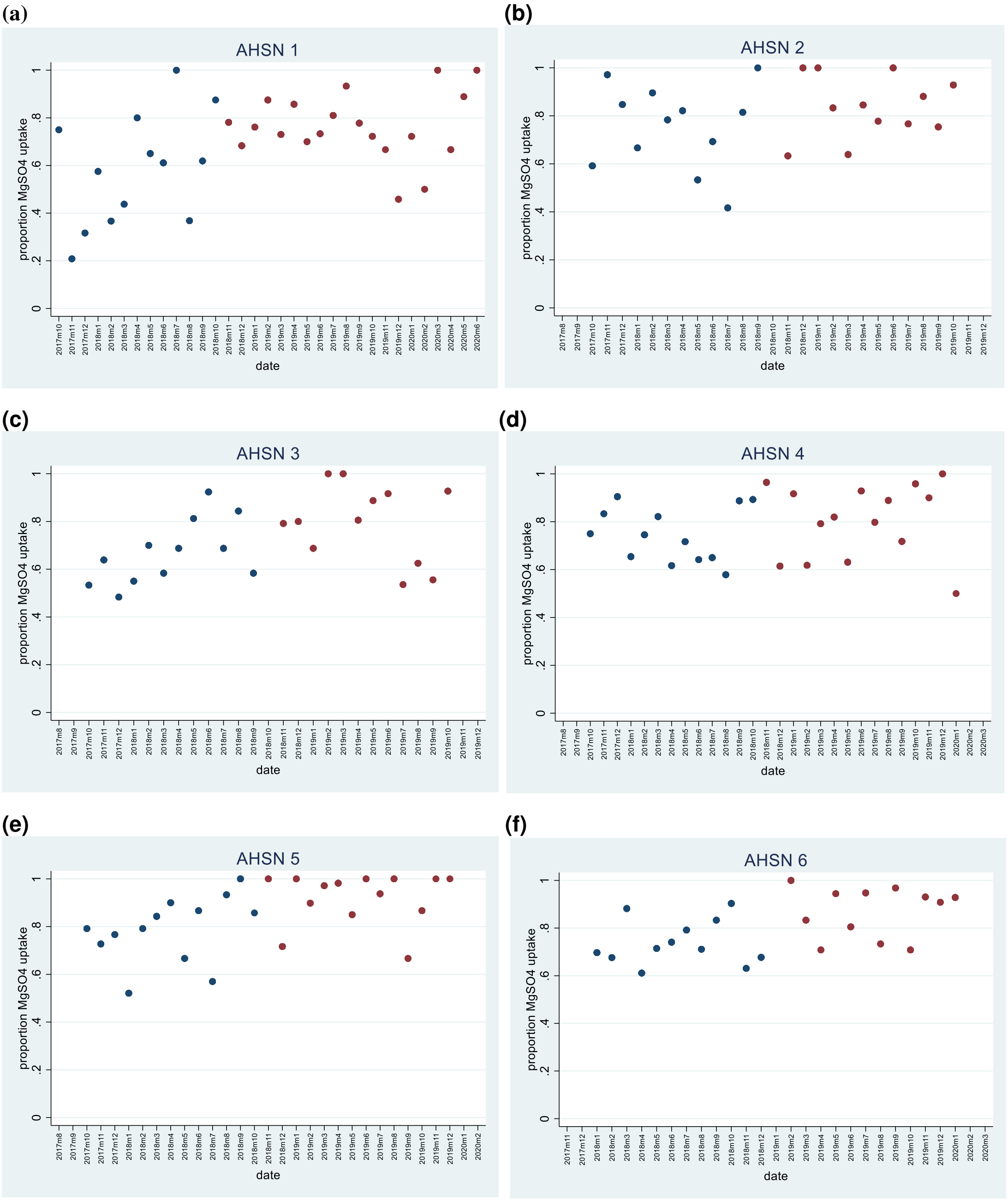

Figure S3. $MgSO_4$ uptake by AHSN*, October 2017 – June 2020 (cont.)

● Pre-implementation periods ● Post-implementation period (including NPP start date)

(h)

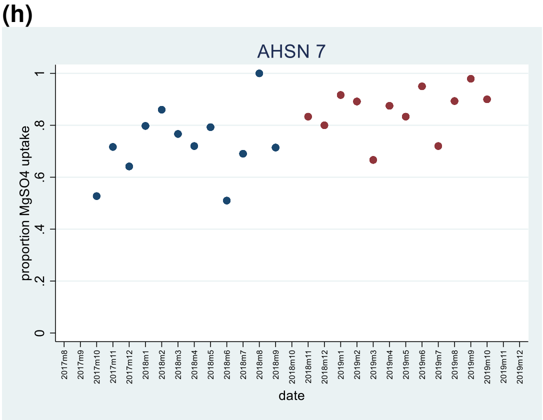

(i)

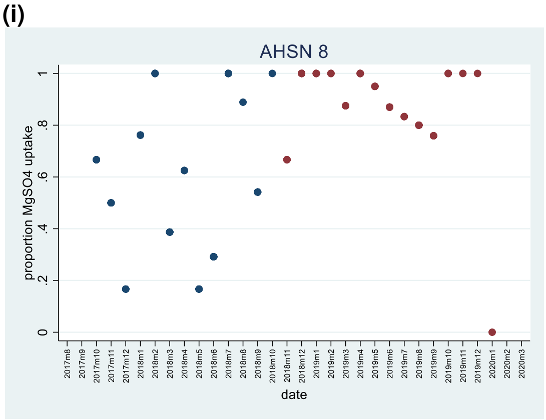

(j)

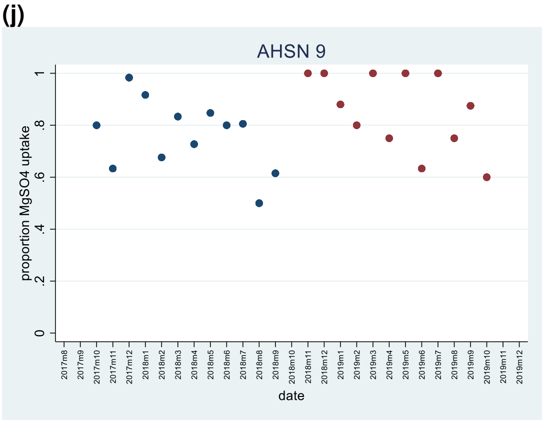

(k)

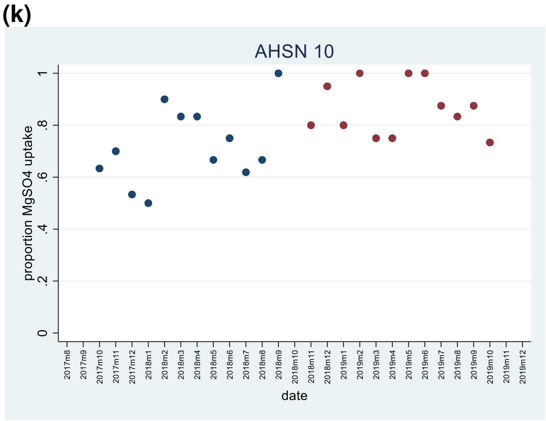

(l)

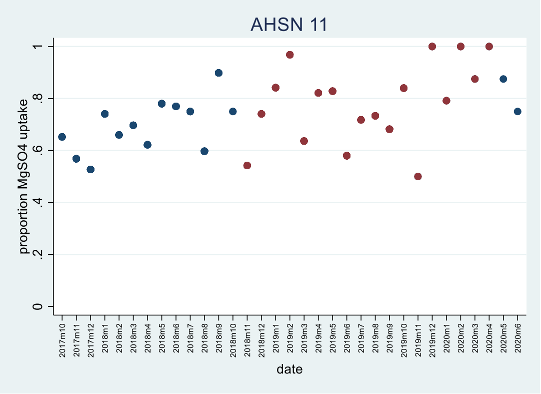

(m)

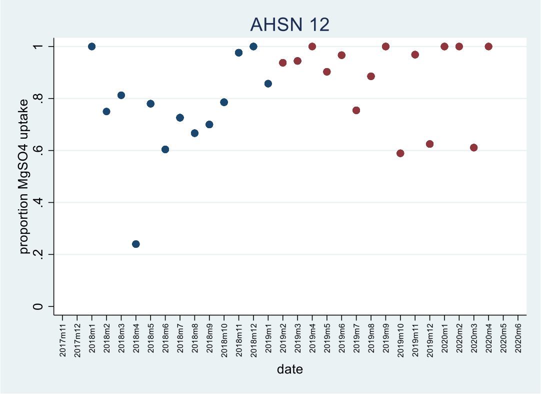

Edwards HB, *et al. Arch Dis Child Fetal Neonatal Ed* 2023; 108:342–347. doi: 10.1136/archdischild-2022-324579

Figure S3. $MgSO_4$ uptake by AHSN*, October 2017 – June 2020 (cont.)

● Pre-implementation periods ● Post-implementation period (including NPP start date)

(n) (o)

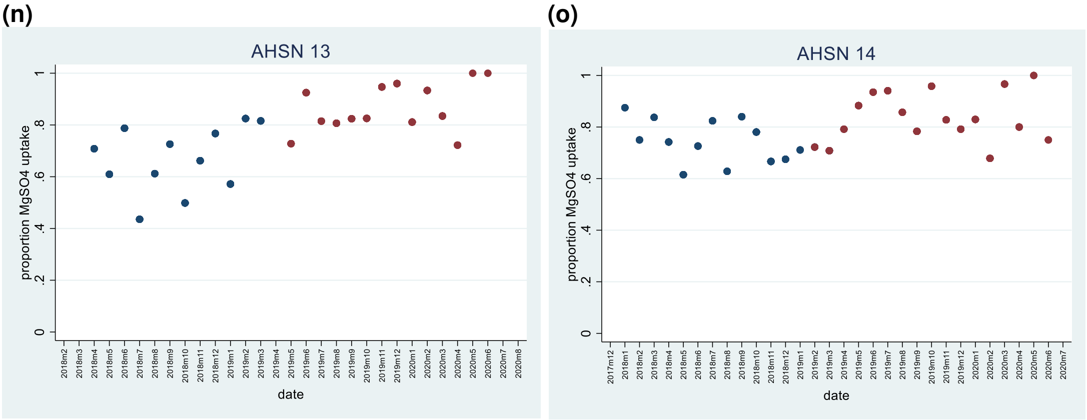

*One AHSN was comprised of pilot sites for the toolkit development and was not included in this study

Edwards HB, *et al. Arch Dis Child Fetal Neonatal Ed* 2023; 108:342–347. doi: 10.1136/archdischild-2022-324579

Trend in missing $MgSO_4$ data over time in maternity units in England

Figure S4. Trend in missing $MgSO_4$ data over time, October 2017 – June 2020

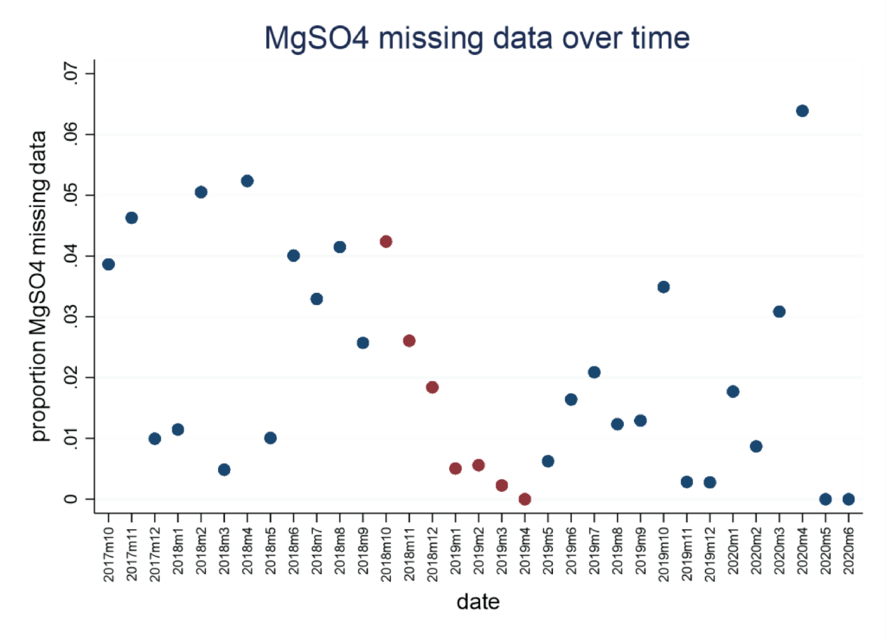

• Pre- or post-implementation periods • NPP implementation start dates

Figure S5. Trend in missing MgSO4 data over time, January 2014 – June 2020

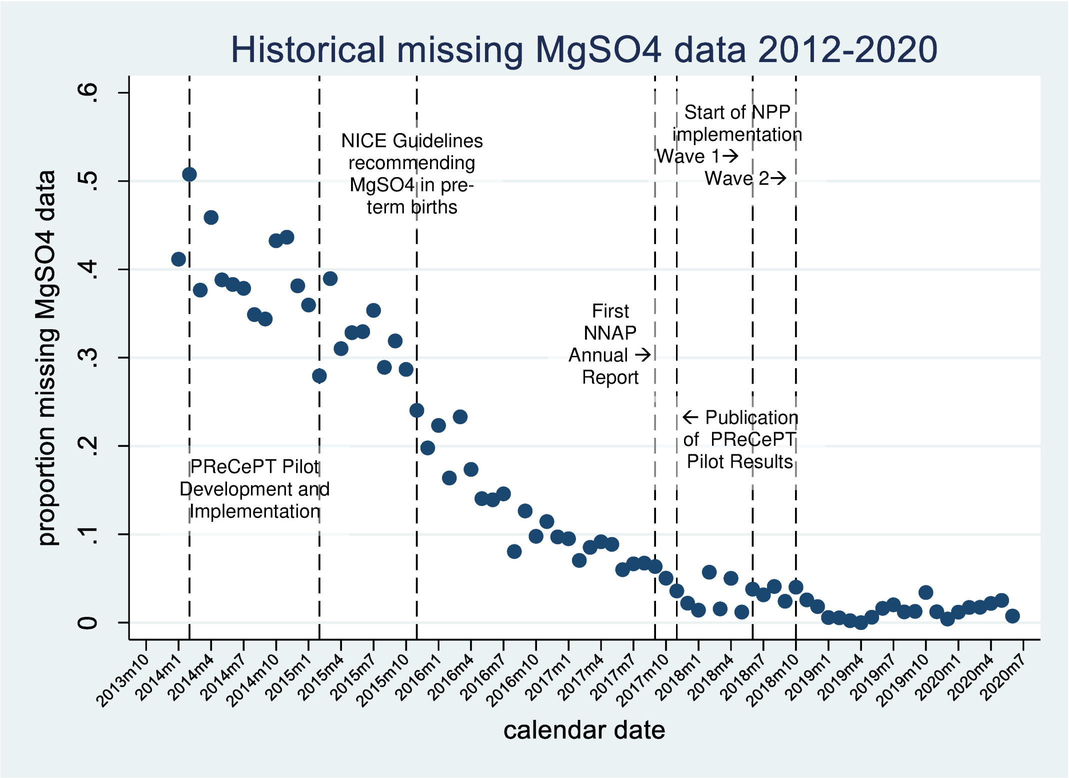

Economic evaluation

Table S2. Estimated lifetime costs and QALYs per patient associated with MgSO4 treatment†

Type of birth	Perspective	Method	Cost, £*	Δcost, £*	QALYs	ΔQALYs
Imminent	Societal	$MgSO_4$	61,971	-23,690	26.6	0.3
		No $MgSO_4$	85,661		26.3	
Threatened	Societal	$MgSO_4$	44,068	-15,964	26.7	0.2
		No $MgSO_4$	60,032		26.5	

† Based on Bickford et al (8)
*Cost estimates were converted to Pounds Sterling and inflated to 2019 prices

Edwards HB, *et al. Arch Dis Child Fetal Neonatal Ed* 2023; 108:342–347. doi: 10.1136/archdischild-2022-324579

Table S3. Point estimates, probability distributions, and source of parameter estimates used in the probabilistic analysis

Parameter	Statistics	Combined Estimates	SCU/HDU Estimates	NICU Estimates	Probability distribution	Source
Type of birth						
Imminent	n (%)	971 (38%)	336 (36%)	635 (39%)	Beta distribution	NNRD data
Threatened	n (%)	1573 (62%)	598 (64%)	975 (61%)	Beta distribution	NNRD data
Probability $MgSO_4$ treatment (yes) – Baseline period						
$MgSO_4$ yes - Imminent	n (%)	679 (74%)	228 (66%)	451 (80%)	Beta distribution	NNRD data
$MgSO_4$ yes - Threaten	n (%)	1311 (73%)	432 (64%)	879 (78%)	Beta distribution	NNRD data
Effectiveness NPP						
$MgSO_4$ yes - Imminent	OR (se)	1.09 (0.03)	1.10 (0.04)	1.07 (0.03)	LogNormal	Logistic regression
$MgSO_4$ yes - Threaten	OR (se)	1.05 (0.03)	1.05 (0.04)	1.04 (0.03)	LogNormal	Logistic regression
Health utility (QALY)						
$MgSO_4$ yes - Imminent	Mean (se*)	0.3 (0.06)	0.3 (0.06)	0.3 (0.06)	Beta distribution	Bickford et al [1]
$MgSO_4$ yes - Threaten	Mean (se*)	0.2 (0.04)	0.2 (0.04)	0.2 (0.04)	Beta distribution	Bickford et al [1]
Lifetime costs						
$MgSO_4$ yes - Imminent	Mean (se*)	£-23,690 (-4,738)	£-23,690 (-4,738)	£-23,690 (-4,738)	Gamma distribution	Bickford et al [1]
$MgSO_4$ yes - Threaten	Mean (se*)	£-15,964 (-3,193)	£-15,964 (-3,193)	£-15,964 (-3,193)	Gamma distribution	Bickford et al [1]
Implementation costs						
$MgSO_4$ yes - Imminent	Mean (sd)	£221 (341)	£473 (500)	£95 (44)	Gamma distribution	NNRD data
$MgSO_4$ yes - Threaten	Mean (sd)	£325 (471)	£553 (701)	£99 (45)	Gamma distribution	NNRD data
$MgSO_4$ no - Imminent	Mean (sd)	£259 (470)	£630 (596)	£97 (56)	Gamma distribution	NNRD data
$MgSO_4$ no - Threaten	Mean (sd)	£360 (603)	£602 (765)	£106 (77)	Gamma distribution	NNRD data

*Standard Errors were assumed to be 20% if their point estimate

Edwards HB, *et al. Arch Dis Child Fetal Neonatal Ed* 2023; 108:342–347. doi: 10.1136/archdischild-2022-324579

Table S4. Mean lifetime QALYs and costs per baby by type of birth and trial arm

	**Before NPP**					**Afer NPP**				
	**Imminent**		**Threatened**			**Imminent**		**Threatened**		
	**$MgSO_4$ Yes**	**$MgSO_4$ No**	**$MgSO_4$ Yes**	**$MgSO_4$ No**	**Total**	**$MgSO_4$ Yes**	**$MgSO_4$ No**	**$MgSO_4$ Yes**	**$MgSO_4$ No**	**Total**
**Status distribution**										
Combined	28%	10%	45%	17%	100%	31%	7%	47%	15%	100%
SCU/HDU units	24%	12%	41%	23%	100%	26%	10%	43%	21%	100%
NICU units	31%	8%	47%	13%	100%	34%	6%	49%	11%	100%
**Lifetime costs**										
Combined	61,971	85,661	44,068	60,032	55,917	61,971	85,661	44,068	60,032	54,982
SCU/HDU units	61,971	85,661	44,068	60,032	57,129	61,971	85,661	44,068	60,032	56,231
NCIU units	61,971	85,661	44,068	60,032	55,155	61,971	85,661	44,068	60,032	54,298
**Implementation costs**										
Combined	0	0	0	0	0.00	221	325	259	360	267
SCU/HDU units	0	0	0	0	0.00	473	630	553	602	550
NICU units	0	0	0	0	0.00	95	97	99	106	98
**QALYs**										
Combine	26.60	26.30	26.70	26.50	26.60	26.60	26.30	26.70	26.50	26.61
SCU/HDU units	26.60	26.30	26.70	26.50	26.58	26.60	26.30	26.70	26.50	26.59
NICU units	26.60	26.30	26.70	26.50	26.61	26.60	26.30	26.70	26.50	26.62

Edwards HB, *et al. Arch Dis Child Fetal Neonatal Ed* 2023; 108:342–347. doi: 10.1136/archdischild-2022-324579

Table S4. Deterministic Analysis Results of the NPP cost-effectiveness

NPP	Combined	SCU/HDU units	NICU units
Incremental implementation costs, £	267	550	98
Incremental lifetime costs, £	-934	-897	-857
Incremental total costs, £	-667	-347	-759
Incremental QALYS	0.01	0.01	0.01
Net Monetary Benefit*, £	903	574	975

*We used a willingness-to-pay threshold of £20,000 per QALY

Edwards HB, *et al. Arch Dis Child Fetal Neonatal Ed* 2023; 108:342–347. doi: 10.1136/archdischild-2022-324579

Figure S6. Cost-effectiveness plane of National PreCePT Programme

The graph displays results of Monte Carlo simulations with 10 000 iterations using the value ranges and distributions presented in Table S3. The horizontal axis represents the effect measures in Quality Adjusted Life Years (QALYs) for National PreCePT Programme; and the vertical axis represents the cost. Datapoints falling the top right quadrant indicate that the National PreCePT Programme is effective and costly. Datapoints falling bottom right quadrant indicate that National PreCePT Programme is effective and cost saving.

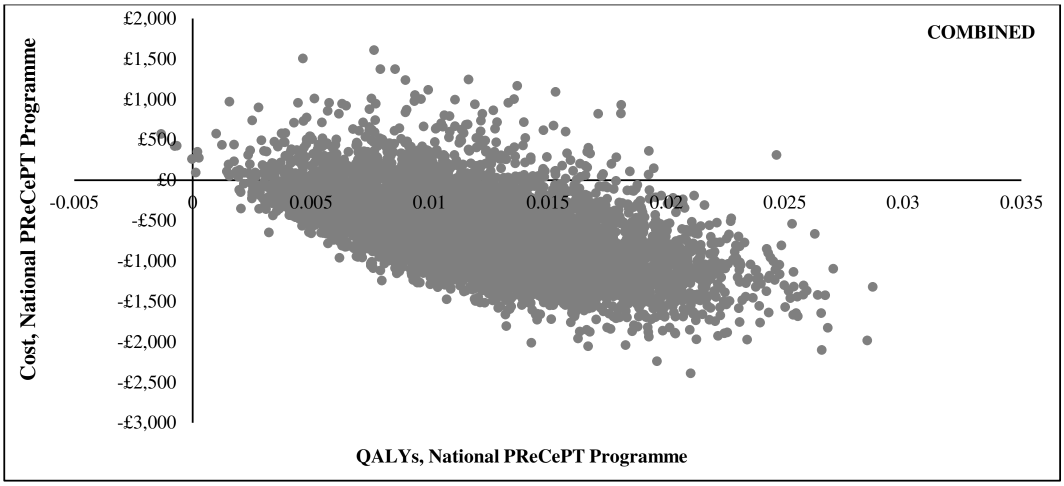

Figure S7. Cost-effectiveness plane of National PreCePT Programme for SCU/HDU units

The graph displays results of Monte Carlo simulations with 10 000 iterations using the value ranges and distributions presented in Table S3 for SCU/HDU units. The horizontal axis represents the effect measures in Quality Adjusted Life Years (QALYs) for National PreCePT Programme; and the vertical axis represents the cost. Datapoints falling the top right quadrant indicate that the National PreCePT Programme is effective and costly. Datapoints falling bottom right quadrant indicate that National PreCePT Programme is effective and cost saving.

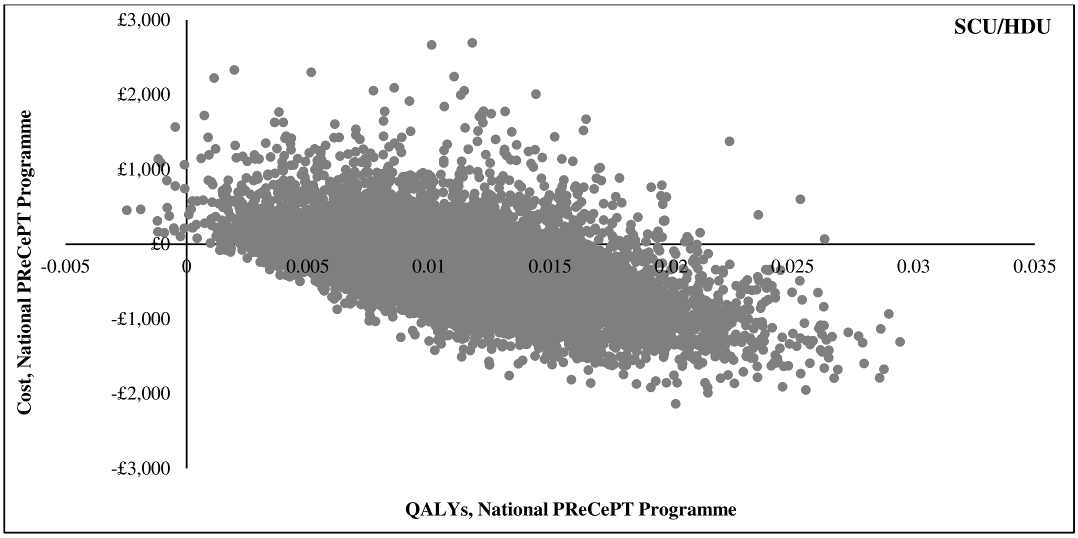

Figure S8. Cost-effectiveness plane of National PreCePT Programme for NICU units

The graph displays results of Monte Carlo simulations with 10 000 iterations using the value ranges and distributions presented in Table S3 for NICU units. The horizontal axis represents the effect measures in Quality Adjusted Life Years (QALYs) for National PreCePT Programme; and the vertical axis represents the cost. Datapoints falling bottom right quadrant indicate that National PreCePT Programme is effective and cost saving.

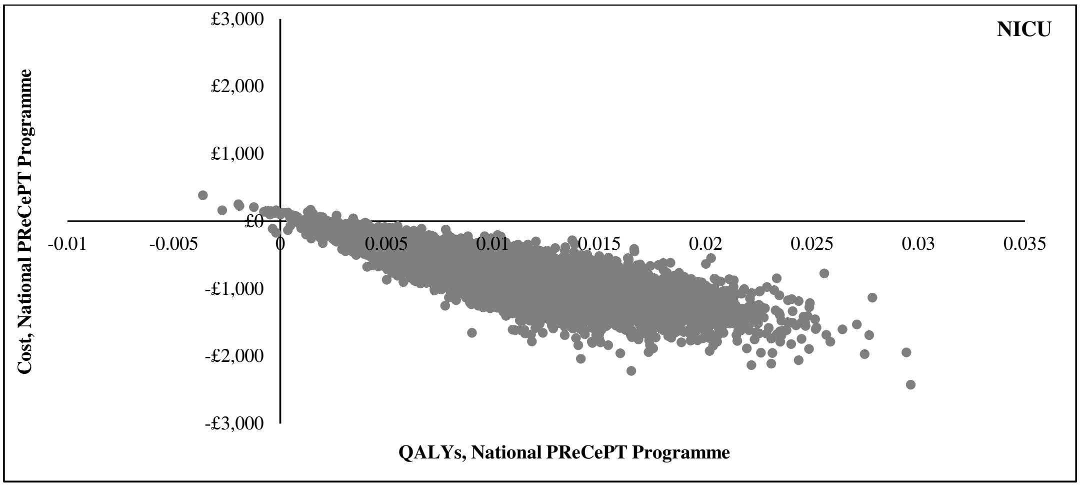

Edwards HB, *et al. Arch Dis Child Fetal Neonatal Ed* 2023; 108:342–347. doi: 10.1136/archdischild-2022-324579

Figure S9. Cost-effectiveness acceptability curve of National PreCePT Programme

The curve shows the probability of National PreCePT Programme being cost-effective at different cost-effectiveness threshold values.

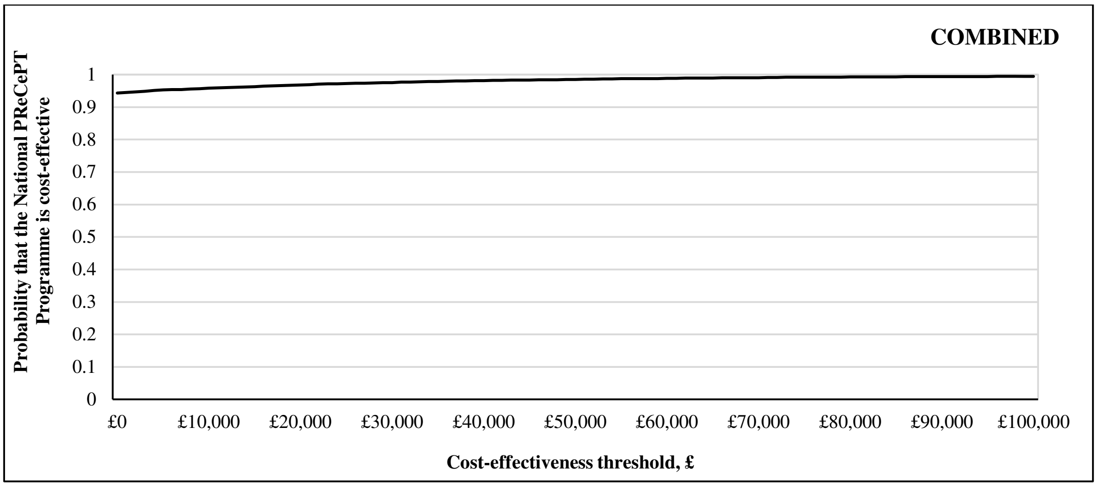

Figure S10. Cost-effectiveness acceptability curve of National PreCePT Programme for SCU/HDU units

The curve shows the probability of National PreCePT Programme being cost-effective at different cost-effectiveness threshold values for SCU/HDU units.

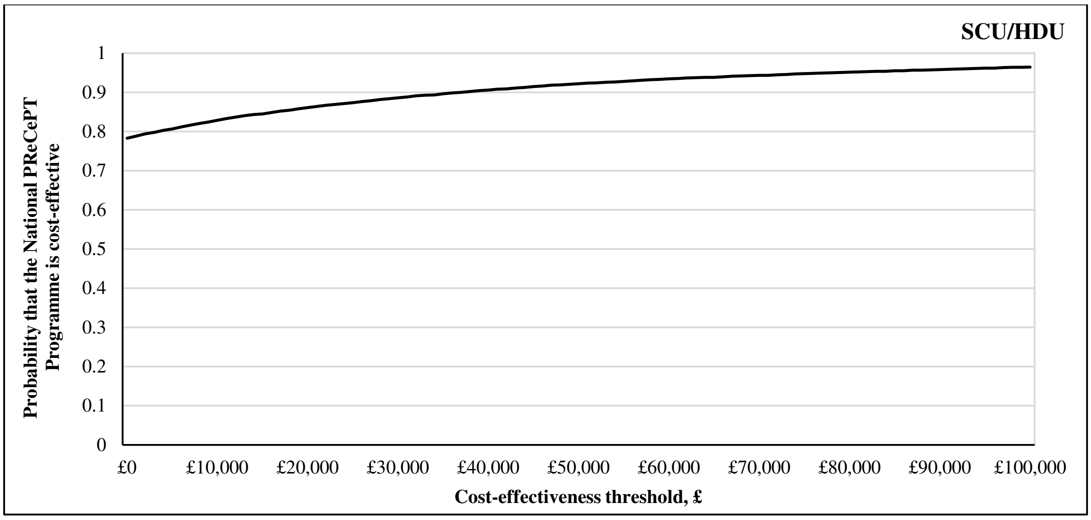

Edwards HB, *et al. Arch Dis Child Fetal Neonatal Ed* 2023; 108:342–347. doi: 10.1136/archdischild-2022-324579

Figure S11. Cost-effectiveness acceptability curve of National PreCePT Programme for NICU units

The curve shows the probability of National PreCePT Programme being cost-effective at different cost-effectiveness threshold values NICU units.

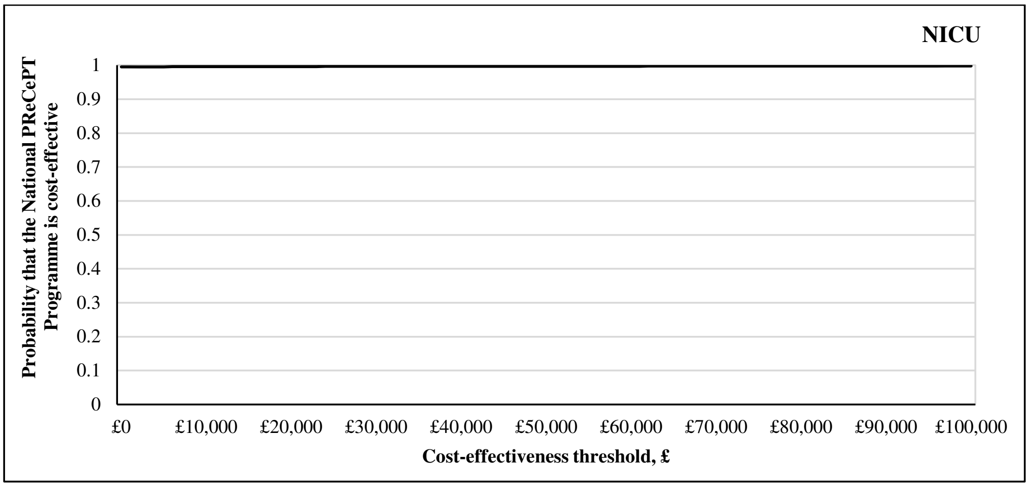

1. Bickford CD, Magee LA, Mitton C, et al. Magnesium sulphate for fetal neuroprotection: a cost-effectiveness analysis. *BMC Health Services Research* 2013; **13**: 527.
